# Inter-Individual Differences in Exercise Responses in Alzheimer’s Disease

**DOI:** 10.1093/geroni/igaa057.608

**Published:** 2020-12-16

**Authors:** Fang Yu, Dereck Salisbury, Michelle Mathiason

**Affiliations:** 1 Arizona State University, Minneapolis, Minnesota, United States; 2 University of Minnesota, Minneapolis, Minnesota, United States; 3 University of Minnesota, Minneapolis, United States

## Abstract

Aerobic exercise is widely supported as a disease-modifying treatment for Alzheimer’s disease (AD) in animal models; however, its effects on cognition have been mixed in human studies, which may be attributable to inter-individual differences in aerobic fitness and cognitive responses to aerobic exercise. This study evaluated inter-individual differences in aerobic fitness and cognitive responses to 6-month aerobic exercise in participants with AD dementia by secondarily analyzing the FIT-AD Trial data. Aerobic fitness with the shuttle walk test (SWT), 6-minute walk test (6MWT), and maximal oxygen consumption (VO2max) from cycle-ergometer exercise test, and cognition with the AD Assessment Scale–Cognition (ADAS-Cog). Inter-individual differences were calculated as the differences in the standard deviation of 6-month change (SDR) in outcomes between the intervention and control groups. The sample size was 78 (77.4±6.3 years old, 15.7±2.8 years of education, 41% women). VO2max was available in 26 participants (77.7±7.1 years old, 14.8±2.6 years of education, 35% women). The results show that the SDR was 37.0, 121.1, 1.7, and 2.3 for SWT, 6MWT, VO2max, and ADAS-Cog, respectively, but there were no statistically significant differences between the intervention and control groups in these measures over six months. Our results indicate that inter-individual differences exist in aerobic fitness and cognitive responses to aerobic exercise in AD, which contributed to the favorable, but not statistically significant between-group differences in aerobic fitness and cognition. To conclude, our study is the first to demonstrate inter-individual differences in the responses to aerobic exercise in AD dementia using SDR.

